# Do post-reproductive aged females promote maternal health? Preliminary evidence from historical populations

**DOI:** 10.1093/emph/eox012

**Published:** 2017-08-23

**Authors:** Alison Gemmill, Ralph Catalano

**Affiliations:** 1 Department of Demography, University of California, Berkeley, 2232 Piedmont Ave, Berkeley, CA 94720, USA; 2 School of Public Health, University of California, Berkeley, CA 94720, USA

**Keywords:** grandmothers, maternal health, intergenerational transfers, historical demography, alloparenting, collective breeding

## Abstract

**Background and Objectives:**

Much literature argues that natural selection conserved menopause and longevity in women because those who stopped childbearing helped bolster daughters’ fertility and reduce infant mortality among grandchildren. Whether the presence of grandmothers ever improved fitness sufficiently to affect longevity via natural selection remains controversial and difficult to test. The argument underlying the grandmother and associated alloparenting literature, however, leads us to the novel and testable prediction that the presence of older women in historical societies could have affected population health by reducing lethality associated with childbearing.

**Methodology:**

Using historical life table data from four societies (Denmark, England and Wales, France and Sweden), we test the hypothesis that death rates among women initiating childbearing declined when the societies in which they were embedded included unexpectedly high frequencies of older women. We use time series analysis to measure the extent to which the observed likelihood of death among women aged 20–24 differs from statistically expected values when the number of older women grows or declines.

**Results:**

In three of the four countries examined, we find an inverse relationship between the frequency of post-reproductive females in the population and odds of mortality among females at the peak of childbearing initiation.

**Conclusions and Implications:**

Results suggest that the presence of older women in a population may enhance population health by reducing mortality among women who face high risk of maternal death, although additional research is needed to determine if this relationship is causal.

## Teaser

Using historical data, the authors find that an unexpected surplus of older females is inversely associated with mortality among females initiating childbearing. These results suggest that the presence of older women may have enhanced population health by reducing mortality among women who face high risk of maternal death, although additional research is needed to determine if this relationship is causal.

## INTRODUCTION

The argument that natural selection conserved menopause and long life in women because those who stopped bearing children helped bolster daughters’ fertility and reduce infant mortality among grandchildren remains controversial [1, 2]. Most, but not all, empirical tests of this ‘grandmother hypothesis’ find reduced infant mortality and improved lifetime reproductive success associated with the presence of grandmothers [3–7]. Other research, moreover, describes breeding strategies in which persons other than parents provide instrumental and other support to mothers that helps infants survive [8]. These ‘alloparents’, commonly post-reproductive women, provide both supplemental care of offspring and reduce the workload of mothers, usually by provisioning food or assisting with domestic labor [5, 6, 9]. Critics, however, claim that the hypothesized fitness benefits reported in the grandmother and alloparenting literature appear too small to have affected the longevity of modern women [10]. These criticisms may detract from the appeal of the grandmother and alloparenting hypotheses as explanations of the evolutionary origins of menopause and long life in women, but they lead us to propose that the presence of older women might affect population health through mechanisms other than reducing infant mortality among direct descendants. Here we elaborate on this proposition and explore historical data for signals of its accuracy.

The grandmother and alloparenting literature assumes that the health benefits of post-reproductive women in the population accrue to infants and children. We, however, know no reason why the health of mothers of these infants and children would not also benefit, particularly before the advent of medical and public health interventions that reduced the high morbidity and mortality associated with pregnancy and childbirth. In 19th century rural Sweden, for example, childbearing caused approximately 40–45% of deaths occurring to women 20–34 years of age [11]. Women initiating childbearing exhibit higher risk of maternal death and disability than those bearing second and later children [12–14]. Older women, however, had both traditional and experiential knowledge about pregnancy and parturition that plausibly mitigated the inherent mortality risks involved in childbearing [15]. A traditional knowledge system of maternal health and practices serves an important function, since humans, compared to other primates, generally require skilled assistance at birth [16]. Taken together, the direct transfers of knowledge and assistance from older to younger women should have reduced morbidity and mortality attributable to childbirth.

No one to our knowledge has previously argued, let alone tested, that the presence of older women in historical societies could have affected population health by reducing lethality associated with childbearing. Having made that argument, we explore high quality historical data from four societies in search of at least one signal it intuitively implies. More specifically, we test the hypothesis that death rates among women initiating childbearing declined when the societies in which they were embedded included unexpectedly high frequencies of older women. As such, our test assumes, as implied by the more generalized alloparenting and collective breeding literature, that all older women, not just direct kin, may serve as potential benefactors [17]. We do not offer this test as a source of strong evidence for our argument, but rather as an attempt to determine if associations implied by the argument appear and thereby warrant further investing in confirmatory tests.

## METHODOLOGY

### Data

All data for our tests come from the Human Mortality Database that includes life table and mortality data from countries with dependable and virtually complete vital statistics [18]. We used data from Denmark, England and Wales, France and Sweden because the time series for these 4 societies started early enough to give us the minimum 50 cases (i.e. consecutive calendar years) thought necessary to conduct the time-series modeling described below [19].

We choose 1913 as our end year because World War I and, subsequently, the Great Flu of 1918/19 induced perturbations in fertility and mortality that likely persisted until the onset of medical interventions that dramatically reduced maternal mortality in the 20th century [20, 21]. Using 1913 as our test end date yielded time series for Denmark of 79 years (starting in 1835), for England and Wales of 73 years (starting in 1841), for France of 98 years (i.e. starting 1816) and for Sweden of 163 years (i.e. starting 1751).

### Variables

We constructed our independent variable from the annual number of women aged 50 through 69 measured in 1000’s. The literature suggests that most women in these societies would have passed menopause by age 50 during our test period [22]. We further reasoned that by age 70, many women became consumers rather than providers of care. We inferred this from the fact that death rates among women in our four-test societies began to increase rapidly at age 70 for all cohorts observed during our test period.

Consistent with estimates of the modal maternal age at first birth in 18th and 19th century Western Europe [23, 24], we used the annual number of deaths per 1000 women 20–24 years old as our dependent variable. We transformed this death rate to natural logarithms to reduce the effect of outliers on our estimations and to allow us to express any significant findings in the metric of percent change in the likelihood of death associated with changes in the number of older women in the population.

### Analyses

Observational tests such as ours essentially determine whether the dependent variable moves away from its statistically expected values when the ‘dose’ of the independent variable increases (or decreases). The units of analysis in our four tests consist of years characterized by the number of women aged 50 through 69 (in 1000 s) and the death rate (per 1000) among 20–24-year-old women. Our tests measure the extent to which the observed death rates for the four test societies differ from statistically expected values when the number of older women grows or declines. Our hypothesis predicts an inverse relationship.

Since the seminal work of Galton [25], tests of association have typically assumed that the expected value of a variable is its mean. Variables measured over time, however, often violate this assumption because they exhibit ‘autocorrelation’ in the form of secular trends, cycles, or the tendency to remain elevated or depressed, or to oscillate, after high or low values. The expected value of such series is not the mean of all observations but rather the value predicted by autocorrelation. Researchers dating to Fisher [26] have solved this problem by using time-series modeling to arrive at the expected values of autocorrelated series. We used a widely disseminated type of such modeling to arrive at the expected values of our dependent variable. The method, devised by Box and Jenkins [27], identifies which of a large family of models best describes a time series. Metaphorically, the method assumes that the series passed through an unobserved ‘filter’ that imposed autocorrelation. The procedure uses mathematical ‘signatures’ to narrow the likely filters to a few and then applies estimates of ‘fit’ to identify the most likely candidate. The differences between the values predicted by the model (i.e. the expected values) and the observed series approximate the values that passed through the filter. They meet the assumptions of traditional tests of association because they are independent of each other (i.e. exhibit no autocorrelation), their expected value equals their mean (i.e. 0) and they exhibit constant variability over time. Removing autocorrelation from our dependent variable has the added benefit of precluding type I errors resulting from trends and cycles that death rates among women aged 20 through 24 might share with the number of post-reproductive aged women in our test societies.

We also structured our tests to reduce the threat of the most intuitive rival hypothesis—that an association arises between our variables because exogenous shocks affect mortality among women regardless of age. A pathogenic shock, unusually bad weather, for example, could cause higher than expected death rates among women aged 20–24 and reduce the population of older women below expected levels. Unusually benign weather could, obversely, lower the death rate among both older and younger women and induce a spurious association. We reduce the likelihood of such ‘general mortality’ effects by including period life expectancy among all women as a covariate in our test. This variable essentially estimates the average age of females who die in a year, and its variation over time indicates shocks that cause death to occur more or less frequently.

Our test proceeded through the following steps.

1. Separately for Denmark, England and Wales, France and Sweden, we regressed the natural logarithm of the death rate (per 1000) among women aged 20 through 24 on annual period life expectancy for females. This step removes variation in the former shared with the latter and thereby rules out the possibility that our test results could arise from a ‘general mortality’ effect.
2. We used Box and Jenkins methods to detect autocorrelation in the residuals of each of the regressions estimated in Step 1 [27]. If we detected any, we expanded the models to include the indicated Box–Jenkins parameters. This step ensures that the results of our test cannot arise from autocorrelation, including trends due to population growth, shared by the death rate among women aged 20 through 24 (adjusted, in step 1, for female period life expectancy), and the number of older women.
3. For each of the 4 societies we estimated the following test equation formed by adding, as a predictor variable, the number of older women to the model developed in Step 2.
  ∇1nP1-Pte=C+ω0∇2nX1t+ω1∇3nX2t+1-θBq1-φBpat

P1-Pte is the natural logarithm of the death rate (in 1000 s) among 20–24-year-old women in year t. $\nabla_{1}^{n}$ is the ‘difference operator’ that indicates P1-Pte, *X*_1_ and *X*_2_ exhibited trends (i.e. non-stationary means) and that we, as required by Box–Jenkins modeling, differenced them (i.e. values at year *t* subtracted from values at year *t *+* n*) before the coefficients were estimated. *X*_1t_ is period life expectancy for females at year *t*. *X*_2t_ is the number of women aged 50 through 69 years (in 1000 s) in year *t*. θ is the moving average parameter of the Box-Jenkins model. φ is the autoregressive parameter of the Box-Jenkins model. a_t_ is the residual of the model at year *t*. We hypothesize that ω_1_ will be significantly less than 0 (*P* < 0.05; one-tailed test).

## RESULTS

**Figure 1.**
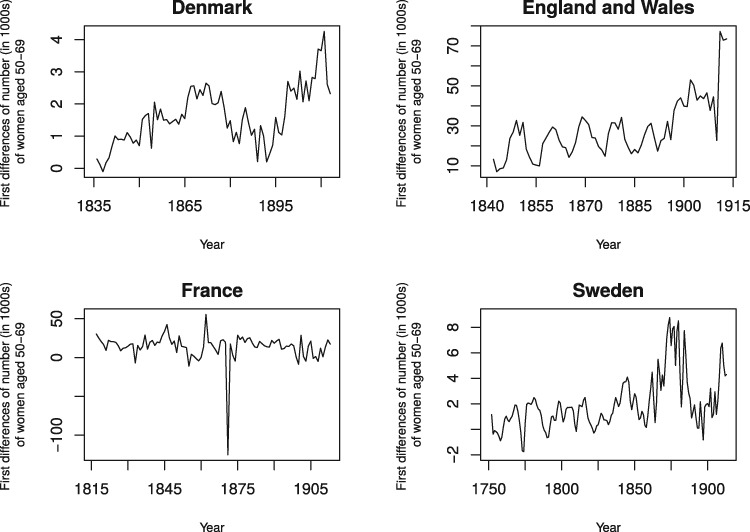
First differences of the number of women (in 1000 s) of women aged 50–69 for Denmark (1835–1913), England and Wales (1841–1913), France (1816–1913) and Sweden (1751–1913)

**Figure 2.**
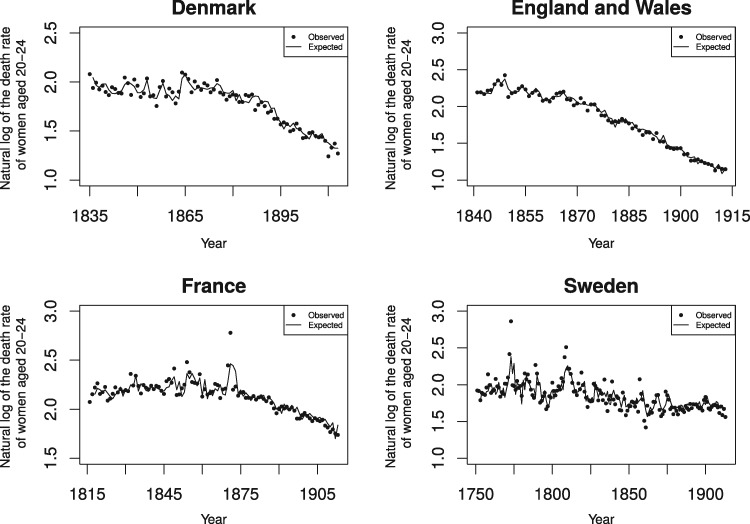
Observed and expected natural log of the death rate among 20–24-year-old women for Denmark (1835–1913), England and Wales (1841–1913), France (1816–1913) and Sweden (1751–1913)

Table 1 shows the mean, standard deviation and range of the 12 time series used in our test. The four panels in Fig. 1 show the first differences of the number of women aged 50 through 69 for the 4 societies over the test period. The four panels of Fig. 2 show the observed and expected (i.e. from period life expectancy and autocorrelation) values of the natural logarithm of the death rate (in 1000 s) among 20–24-year-old women during the test period.

**Table 1.** Means, range and standard deviations of time series used in tests

Country	Variable	Mean	Range	Standard deviation
Denmark (1835–1913)	Death Rate (1000’s) women aged 20–24	6.10	3.46–8.13	1.20
	Female Period Life Expectancy	48.38	39.83–60.11	5.26
	Women aged 60 through 84 (1000’s)	137.64	86.03–211.73	37.16
England and Wales (1841–1913)	Death Rate (1000’s) women aged 20–24	6.64	3.10–11.30	2.27
	Female Period Life Expectancy	45.95	38.14–56.41	4.29
	Women aged 50 through 69 (1000’s)	1934.18	1120.25–3154.10	563.80
France (1816–1913)	Death Rate (1000’s) women aged 20–24	8.56	5.68–16.09	1.48
	Female Period Life Expectancy	43.29	32.42–53.75	4.24
	Women aged 50 through 69 (1000’s)	3118.16	2384.00–3713.70	383.71
Sweden (1751–1913)	Death Rate (1000’s) women aged 20–24	6.48	4.13–17.49	1.53
	Female Period Life Expectancy	44.12	18.79–59.98	7.17
	Women aged 50 through 69 (1000’s)	253.40	141.50–465.56	99.24

Table 2 shows the results of steps 1 through 3 above. Consistent with our hypothesis, the coefficients (i.e. −0.0003, −0.0016 and −0.0226) for the number of post-reproductive women in England and Wales, France and Sweden respectively are significantly (i.e. *P* < 0.01; one-tailed test) less than 0 for all three tests. The coefficient for Denmark (i.e. −0.0016) did not differ from 0.

**Table 2.** Coefficients (standard errors) for test models predicting the natural logarithm of the death rate (1000’s) of women aged 20 through 24

Country	Parameter	Model
Denmark (1835 - 1913)	$n\;in\;\nabla_{1}^{n}$ (from test equation)	*n* = 1
	Female period life expectancy	−0.0244** (0.0032)
	Women 50 through 69 (1000’s)	−0.0016 (0.0015)
	Moving average parameter at t-1	0.6224** (0.0903)
England and Wales (1841 – 1913)	$n\;in\;\nabla_{1}^{n}$ (from test equation)	*n* = 1
	Female period life expectancy	−0.0308** (0.0034)
	Women 50 through 69 (1000’s)	−0.0003** (0.0001)
	Moving average parameter at t-1	0.3198** (0.1162)
France (1816 – 1913)	Constant	2.0513** (0.1774)
	Female period life expectancy	−0.0152** (0.0015)
	Women 50 through 69 (1000’s)	−0.0016** (0.0003)
	Autoregressive parameter at t-1	0.9595** (0.0397
Sweden (1751 - 1913)	Constant	1.8908** (0.0388)
	Female period life expectancy	−0.0169** (0.0018)
	Women 50 through 69 (1000’s)	−0.0226** (0.0083)
	Autoregressive parameter at *t* − 1	0.7566** (0.0540)

* *P* < 0.05; one-tailed test.

** *P* < 0.01; one-tailed test.

As expected, female period life expectancy varied inversely and significantly with death rates among women aged 20 through 24 for all 4 societies. As suggested by the plots in Fig. 2, the longer series of death rates from France and Sweden did not exhibit trend as consistently as the shorter and later series from Denmark and England and Wales, both of which required differencing (i.e. $n\;in\;\nabla_{1}^{n}$ shown in the test equation equals 1).

We put the ‘dose response’ of our discovered associations in context by estimating change in *R*^2^ attributable to changes in the number of older women. The model estimated in the first two steps above accounted for 55, 83 and 66% of the variance in the natural logs of the death rate (in 1000 s) among women aged 20–24 for England and Wales, France and Sweden, respectively. Adding the older women variable to the equation increased *R*^2^ to 59, 88 and 68% for the three countries in the same order.

We can provide additional, if less familiar than change in *R*^2^, context for our results because we transformed our dependent variables to their natural logarithms. Multiplying the coefficients shown in Table 2 for the number of women aged 50 through 69 by the average change (in thousands) of such women yields an estimate of the average effect on death rates among women entering reproductive life in the country. In France, e.g. the average change in the number (in thousands) of older women (i.e. an increase of 13.7 thousand) predicted a 2.1% decline in the death rate among women entering reproductive life (i.e. 0.0016 × 13.7 × 100). To put this estimate in context we note that death rates per 1000 women aged 20 through 24 in France fell about 29% (i.e. from 7.95 to 5.68) over our test period or an average of 0.3% per year.

Replicating these calculations for Sweden suggests that the average change in older women (i.e. an increase of about 2000) would predict a decline in death rates among women aged 20 through 24 of 4.5% (i.e. 0.0226 × 2 × 100) in the average year. The death rate among these women fell from 6.8 (per thousand) in 1751–4.76 in 1913 or about 30% for an average annual drop of 0.18%.

Applying the above logic to England and Wales suggests that the average change (in thousands) of older women (i.e. an increase of about 28.25) would predict a decline in death rates among women aged 20 through 24 of 0.85% (i.e. 0.0003 × 28.25 × 100) in the average year. The death rate among these women fell from 8.9 (per thousand) in 1841–3.2 in 1913 or about 64% for an average annual drop of 0.87%.

Although our argument assumes a contribution of older women to the health of women entering reproductive age, the theory of alloparenting does not preclude that older men also contribute. To explore this possibility, we added annual differences in the number of men aged 50 through 69 (in 1000 s) to our test equations for the three countries in which we rejected the null in our main tests (i.e. England and Wales, France and Sweden) and estimated all coefficients again. Results from France showed that increases in the number of older women remained significantly associated with reductions in death rates among women entering reproductive life while the coefficient for older men did not differ from 0. Results from Sweden showed that including older men and women effectively cancelled each other out implying collinearity in which the numbers of both changed so similarly that we could not reliably estimate the independent association of either. In England and Wales, both coefficients differed significantly from 0 but that for women, consistent with our argument, remained less than 0 while that for men exceeded 0. *Post hoc* explanations of the results from England and Wales include that older women may have invested more effort in older males than in younger females in their social networks.

We did not set out to determine whether the population enjoys health benefits from the presence of older women but rather to test the narrower hypothesis, implied by the alloparenting literature, that death rates among women entering reproductive life would fall when the population of post reproductive women expands. Our data, however, allow us to estimate associations that might motivate other researchers to explore contributions from older women to the health of persons other than women aged 20 through 24. We, for example, used Box–Jenkins methods described above to estimate the association between male period life expectancy at birth (i.e. essentially average age at death) and the number of older women, controlling for autocorrelation, for the three countries in which we rejected the null hypothesis in our main tests. A significant positive association appeared for England and Wales (0.0069; SE = 0.0016) but no association appeared for either France or Sweden.

We also estimated the association between female period life expectancy and the number of older women controlling for the death rate among women aged 20 through 24 and for autocorrelation for the four countries. A significant positive association appeared for Sweden (i.e. 0.0426; SE = 0.0129) implying that an unexpectedly large increase in the number of older women coincided with reduced deaths among women other than those aged 20 through 24. We found no association for England and Wales or France.

## DISCUSSION

In three of the four countries examined, we find an inverse relationship between the frequency of older, post-reproductive females in the population and odds of mortality among females at the peak of childbearing initiation. Since maternal deaths comprise almost half of all deaths in early reproductive-aged women [11], these results appear to support the hypothesis that that an unexpected surplus of older females mitigated risks of maternal death.

The null finding in Denmark deserves further investigation, as any post-hoc explanation would be largely speculative. We note, however, that demographic patterns in the country were largely similar to its European neighbors [28].

Although historical life table data do not allow for identification of mechanisms that account for this relationship, we consider our maternal health narrative, grounded in theory and evidence, to be the most parsimonious explanation of our findings. Older women’s accrual of intellectual capital pertaining to pregnancy and childbirth should benefit women who have yet to experience reproduction. Older women, moreover, have more time available to invest in offspring’s fertility, including direct care during pregnancy and childbirth.

Taken as a group, our secondary tests appear consistent with the argument that older women contribute more to alloparenting than do older men and that women entering reproductive life likely benefit disproportionately from alloparenting. We found little support for any connection between the number of older women in a society and period life expectancy among males or among females other than those aged 20 through 24. We found similarly little support for an inverse correlation between the number older men and death rates among women entering reproductive life.

This study makes at least three contributions to the existing literature. First, using historical life table data allows us to test our hypotheses in societies characterized by levels of maternal mortality believed similar to those found in populations over long periods of human history yet similar to those of contemporary hunter gatherer societies [29]. Second, this study expands prior scholarship on the cooperative breeding hypothesis by testing for associations with mortality among females entering reproduction and specifically invoking salutary effects on maternal health. Third, the work provides additional support for the assumptions underlying inclusive fitness theory, whereby members of a society seek to allocate their resources in such a way that maximizes the fitness of collateral kin [30, 31].

Our analyses cannot rule out all rival hypotheses to our argument based on alloparenting. We note, however, that an unmeasured factor that affected the health of most women could not have spuriously induced our results because including period life expectancy for women in our analyses did not change the results. To induce our finding, an unmeasured factor would have had to similarly affect survival among women 20 through 24 and 50 through 69 years old but have had no effect on period life expectancy for women. We can think of no confounder that would have such a pattern of age-specific mortality among women.

As we noted above in Methods, applying Box-Jenkins analyses to our dependent variables controls for confounders, including population growth, that exhibit trends and other forms of autocorrelation. Variation remaining after adjustment for autocorrelation logically arises from either stochastic variation in the size of the female birth cohorts that aged into the cohorts of older women in our study, or to exogenous shocks that affected death rates among women reaching old age. These exogenous shocks could have occurred when women reached old age, or they could have occurred earlier in life but affected death rates among exposed cohorts upon reaching old age. We further note that the association we found could not arise from the former of these circumstances because we, as noted above, controlled for this possibility by adding female period life expectancy as a covariate. Our findings therefore, most likely arise either from stochastic variation in the size of female birth cohorts that aged into our cohorts of older women, or from exogenous shocks early in life that affected death rates in those cohorts when they reached old age.

Our findings may have implications for modern populations where many women continue to rely on traditional birth attendants for support during pregnancy and childbirth. In many contexts, traditional birth attendants are often older women who are revered for their acquired knowledge of traditions and practices [32]. Moreover, results from maternal and child health interventions suggest that older women should be viewed as an important resource for enhancing the health of reproductive-aged women [33].

**Conflict of interest**: None declared.
